# Dynamic Temporal Modeling of Abdominal Aortic Aneurysm Morphology with Z–SINDy

**DOI:** 10.1101/2025.09.29.25336910

**Published:** 2026-01-05

**Authors:** Joseph A. Pugar, Junsung Kim, Michael Mansour, Nhung Nguyen, Cheong Jun Lee, Hence Verhagen, Ross Milner, Andrei A. Klishin, Luka Pocivavsek

**Affiliations:** 1 Department of Surgery, University of Chicago, Chicago, IL, USA; 2 Harvard Medical School, Harvard University, Boston, MA, USA; 3 Department of Medical Physics, University of Chicago, Chicago, IL, USA; 4 Endeavor Health, Evanston, IL, USA; 5 Department of Vascular Surgery, Erasmus University Medical Center, Rotterdam, the Netherlands; 6 Department of Mechanical Engineering, University of Hawai’i at Mānoa, HI, USA

## Abstract

Abdominal aortic aneurysm (AAA) is characterized by a localized enlargement of the aorta with risk of rupture. Endovascular aneurysm repair (EVAR) alters sac morphology in ways not captured by size alone and typically observed only at sparse follow-up times. We present a physically interpretable, noise-aware framework that models postoperative remodeling dynamics in a low-dimensional state space defined by normalized sac surface area $\left(\tilde{A}\right)$ and the normalized fluctuation in integrated Gaussian curvature $\left(\tilde{\delta K}\right)$. Using Sparse Identification of Nonlinear Dynamics (Z–SINDy), we infer ordinary differential equations governing the temporal evolution of $\left(\tilde{A},\tilde{\delta K}\right)$ for clinically defined cohorts of regressing and stable sacs. The learned models yield class-specific flow fields and fixed points that summarize long-term behavior: regressing sacs converge toward a low-size/low-shape attractor, whereas stable sacs maintain near-constant size with persistently elevated shape. Embedding these dynamics in a Bayesian decision framework enables complementary static (coordinate-based) and dynamic (derivative-based) classifiers. Across cohorts, the dynamic classifier separates outcomes earlier and with higher confidence, demonstrating that rates of change become informative before static anatomical distributions separate. Stress tests injecting spatial noise and imposing realistic, irregular follow-up cadences quantify performance degradation and sensitivity to measurement fidelity and scheduling. Together, these results provide a principled route from interpretable geometric features to individualized, probabilistic forecasts of AAA remodeling post-EVAR and offer actionable guidance for surveillance design.

## Graphical Abstract

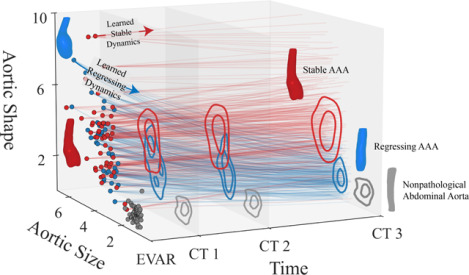

## Introduction

**Figure 1:**
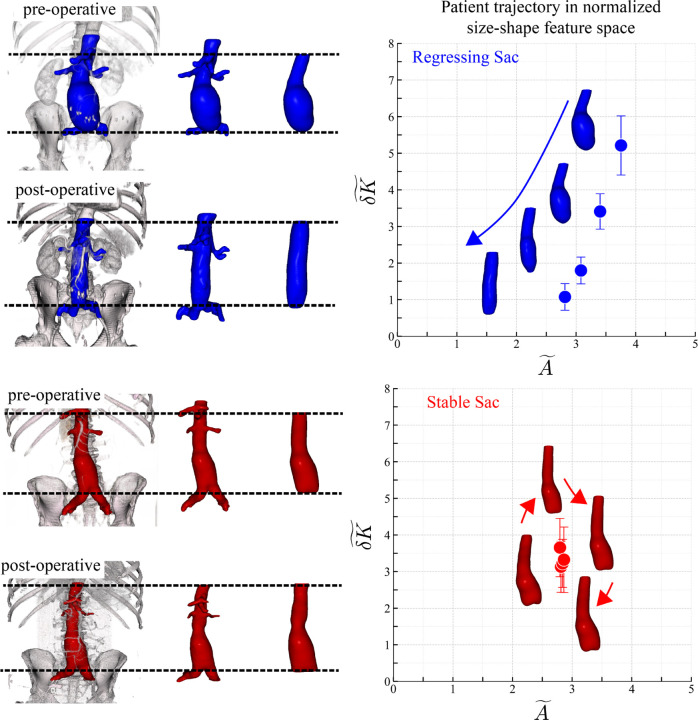
Defining aortic anatomy and mapping trajectories into size–shape space**. Left:** 3D segmentations of AAA scans before and after EVAR. Dashed horizontal lines mark the superior and inferior cut planes used to standardize the analyzed sac segment across time points. **Right:** Each scan is projected into a normalized size–shape space $\left(\tilde{A},\tilde{\delta K}\right)$, where $\tilde{A}$ denotes normalized surface area and $\tilde{\delta K}$ the normalized fluctuation in integrated Gaussian curvature. Points are serial scans connected chronologically; arrows indicate temporal progression. Top row: regressing sac (blue; > 10%/year decrease in surface area). Bottom row: stable sac (red; < 10%/year change). Together, the panels show the workflow—from standardized segmentation via consistent cut planes to quantitative embedding—used to characterize postoperative sac behavior in a common, interpretable feature space.

Abdominal aortic aneurysms (AAA) are localized dilations of the aorta that can develop into a balloon-like *sac* with substantial rupture risk if left untreated. Endovascular aneurysm repair (EVAR) is the dominant minimally invasive intervention used to exclude the sac from arterial flow, promote thrombus formation, and ideally induce sac regression over time (Fig. 1) [1, 2, 3]. Despite widespread adoption, predicting long-term outcomes post-intervention remains challenging [4, 5, 6]. Clinicians primarily rely on size assessments at discrete time points (typically cross-sectional diameter or sac volume) to characterize status [7, 8, 9]. In addition, pathological shape, captured by the fluctuation in integrated Gaussian curvature δK, contains information about disease progression [10].

However, instantaneous anatomical descriptors are often insufficient to infer the underlying trajectory of disease progression. Differentiating a stable post-EVAR sac from one undergoing regression or expansion requires dynamic characterization of morphology over time. The importance of dynamics is reflected in supplemental guidance that characterizes regression by annual rates of maximal diameter change [11], with thresholds such as ≥5–10 mm at 1 year used in practice [4, 12]. Failure to regress at 1 year and sac expansion are associated with higher long-term mortality independent of endoleaks or reinterventions [4, 5], and a “stable” sac carries worse prognosis than a regressing sac [6, 13]. Early sac shrinkage (e.g., ≥10 mm at 1 year) is linked to freedom from fatal adverse events [12], whereas delayed and persistent type II endoleaks correlate with sac growth and adverse late outcomes [14, 15]. The ability to model and anticipate trajectories could inform surveillance frequency, enable early identification of treatment failure, and guide personalized intervention strategies. Substantial regression to ≤40 mm has been associated with very low risk and may permit extended follow-up intervals in selected patients [16].

The dynamics of AAA remodeling are shaped by biomechanical, anatomical, and patient-specific factors. Real-world datasets are limited in size and irregularly sampled, yet they encode latent patterns of morphological change. Rather than reducing outcomes to discrete labels, it is valuable to view post-EVAR behavior as a continuum governed by rates and directions of change in biologically meaningful features. Clinical imaging is sparse: patients may contribute as few as two usable time points (typically one pre- or peri-operative scan and one postoperative scan) and at most about five, separated by months or years. This sparsity—compounded by variation in imaging frequency, segmentation algorithms, and device type—demands models that are robust to noise and heterogeneity and capable of extracting population-level trends from underdetermined individual trajectories. Heterogeneity is further amplified by device-specific effects and repair complexity: most sac regression occurs within the first two years [17]; regression patterns vary across endografts [12]; and complex repairs (fenestrated/branched) exhibit distinct sac dynamics requiring intensified surveillance when growth occurs [18, 19].

Traditional approaches to temporal modeling in clinical science—spanning biostatistics, time-series machine learning [20, 21], and black-box deep learning [22, 21]—are often inadequate here. Linear models underfit nonstationary biological processes, while flexible nonlinear methods risk overfitting noise and spurious fluctuations. These frameworks also rarely accommodate uncertainty from variable sampling intervals or missing data [23, 20, 24]. Black-box models may extract unimportant patterns while overlooking core mechanistic relationships [22, 21]. Effective clinical models must balance complexity and interpretability, capturing coarse-grained system dynamics without overfitting high-dimensional noise. This calls for frameworks that incorporate physical structure, account for data corruption, and remain interpretable to clinicians, particularly when predicting forward trajectories under real-world constraints such as follow-up attrition and irregular scan timing. In parallel, morphovolumetric metrics can be more sensitive than diameter alone for detecting clinically meaningful sac change, underscoring the need to target features that better reflect underlying biomechanics [7].

In this work, we develop a physically interpretable, noise-aware framework for modeling post-EVAR AAA remodeling using a sparse identification approach. Sparse Identification of Nonlinear Dynamics (SINDy) [25, 26] has successfully identified governing equations from synthetic data and reduced-order models, but has seen more limited success with noisy experimental data [27, 28, 29]. We use Z–SINDy [30], a version equipped with uncertainty quantification rooted in statistical mechanics. We infer sparse, interpretable ordinary differential equations (ODEs) governing the temporal evolution of sac surface area A and sac shape δK, features that compactly encode aneurysm morphology. More broadly, our approach provides a general framework for learning and validating low-order dynamical models from sparse, irregularly sampled clinical time series under realistic noise and attrition.

We curated a dataset of 75 patients and 220 CT scans across two clinical cohorts in different countries. Our framework integrates clinical CT time-series data with finite-element–based synthetic up-sampling (FEA) [31] to mitigate sparsity and enhance signal. We demonstrate that Z–SINDy reproducibly identifies dynamic signatures associated with sac regression versus stabilization, provides interpretable steady-state fixed points with clinical meaning, and enables individualized probabilistic forecasts of anatomical evolution. We further examine how sampling frequency, noise, and attrition affect model fidelity, and we propose a principled way to assess confidence in outcome prediction using a Bayesian classification model, thereby aligning physical interpretability with clinical utility.

## Results

### Data curation

**Figure 2:**
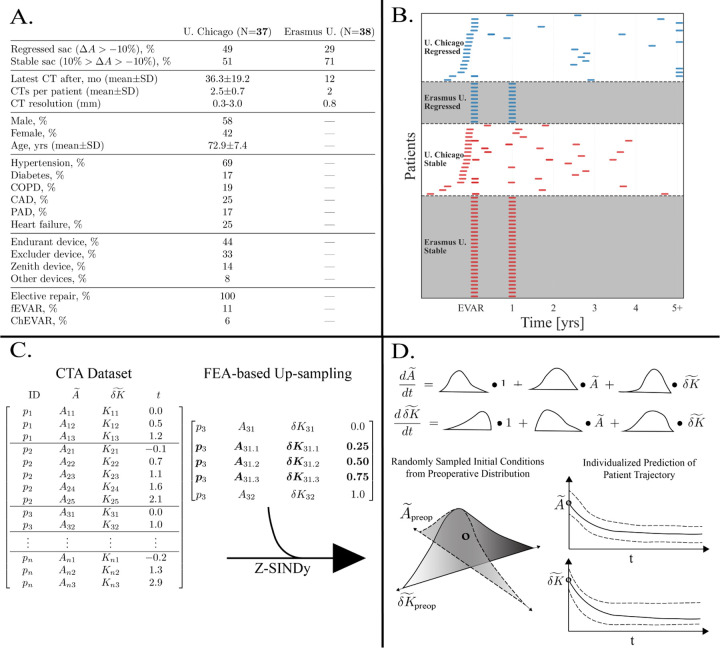
Dataset, sampling, and Z–SINDy pipeline. **A** Demographic and clinical metadata for the two study cohorts (University of Chicago and Erasmus University) used in all analyses. **B** Barcode plot summarizing data-acquisition cadence: each row is a patient; horizontal position is time post-EVAR; ticks indicate CT acquisitions. This view makes temporal sparsity explicit and shows where derivative information for SINDy can be estimated. **C** Input data matrix for modeling, consisting of discrete observations of normalized size–shape variables $\left(\tilde{A},\tilde{\delta K},t\right)$ per patient. Patients contribute a variable number of rows reflecting the sparsity seen in panel B. To reduce sparsity, the true observations are augmented with FEA-based surrogates so that each patient has samples on a 3-month grid between their first and last scan. **D** Schematic of the Z–SINDy methodology. The matrix in panel C is converted into a sparse system of ODEs with coefficient distributions. The ODEs are integrated forward from initial conditions sampled from the preoperative distribution $\left({\tilde{A}}_{\text{preop}},{\tilde{\delta K}}_{\text{preop}}\right)$, yielding probabilistic trajectories for the regressing and stable dynamical models.

We curated a dataset of 75 patients and 220 CT scans across two clinical cohorts from different institutions: the University of Chicago (USA) and Erasmus University Medical Center (the Netherlands), with basic demographic statistics in Fig. 2A. Owing to differences in healthcare systems, the acquisition cadence differs substantially: in the Erasmus cohort each patient typically has one preoperative CT scan immediately before EVAR and one follow-up exactly 12 months later, whereas in the University of Chicago cohort the preoperative scan may precede EVAR by several months and follow-ups are irregular in both spacing and number (Fig. 2B). The scan-based data points were upsampled with the FEA procedure to increase data density (Fig. 2C).

### Modeling post-EVAR dynamics with Z–SINDy

**Figure 3:**
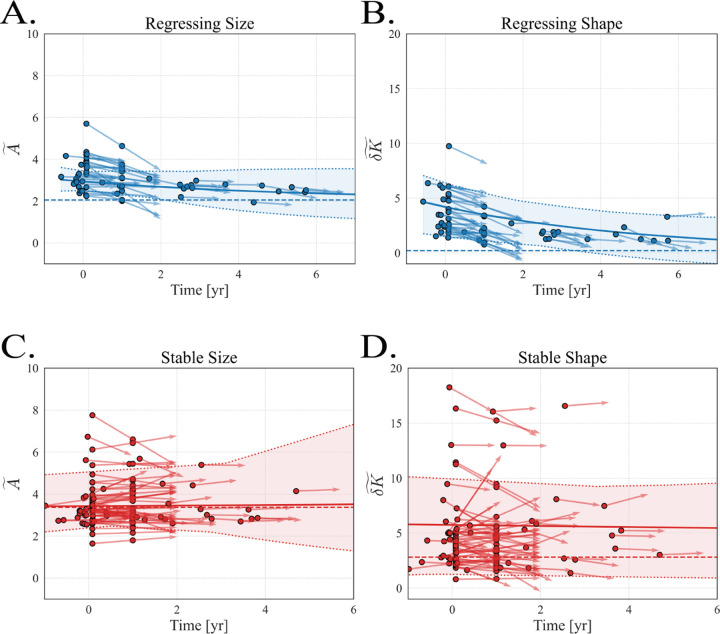
Z–SINDy trajectories for regressing and stable sacs in normalized size–shape space. Panels show the evolution of normalized surface area $\tilde{A}(t)$ and normalized fluctuation in integrated Gaussian curvature $\tilde{\delta K}(t)$ over time t (years since EVAR). **A**–**B** (blue): regressing model; **C**–**D** (red): stable model. Points are observed scan values; faint lines are individual Z–SINDy trajectory realizations obtained by integrating the learned ODEs from population-level initial conditions while sampling coefficient distributions. Bold lines are mean trajectories; shaded regions indicate ensemble spread induced by coefficient uncertainty. Dashed horizontal lines mark steady-state (fixed-point) solutions computed from the mean ODEs. Mean fixed points in $\left(\tilde{A},\tilde{\delta K}\right)$ are approximately (2.1, 0.2) for the regressing model and (3.4, 2.8) for the stable model. Panel assignments: **A** $\tilde{A}(t)$ (regressing), **B** $\tilde{\delta K}(t)$ (regressing), **C** $\tilde{A}(t)$ (stable), **D** $\tilde{\delta K}(t)$ (stable).

We first asked whether coarse-grained dynamics of postoperative sac remodeling can be captured by low-order, interpretable models in the normalized size–shape space $\left(\tilde{A},\tilde{\delta K}\right)$:

$$\frac{d\tilde{A}}{dt}=f\left(\tilde{A},\tilde{\delta K};\Xi \right),\;\frac{d\tilde{\delta K}}{dt}=g\left(\tilde{A},\tilde{\delta K};\Xi \right),$$

where Ξ are model coefficients inferred from the training data. We fitted Z–SINDy models separately to subjects clinically labeled regressing and stable to obtain posterior distributions of coefficients (Fig. 2D). We then resampled both coefficients and initial conditions and integrated the equations forward, yielding ensembles of trajectories with uncertainty bands around the mean solutions (Fig. 3). For the regressing cohort (blue), both $\tilde{A}(t)$ and $\tilde{\delta K}(t)$ decline monotonically toward a low-size/low-shape fixed point; for the stable cohort (red), $\tilde{A}(t)$ remains approximately constant while $\tilde{\delta K}(t)$ remains elevated relative to the regressing group. Fixed-point analysis of the mean ODEs gives $\left({\tilde{A}}^{*},{\tilde{\delta K}}^{*}\right)\approx (2.1,0.2)$ for the regressing model and ≈ (3.4, 2.8) for the stable model (Fig. 3, dashed lines), providing compact, clinically interpretable summaries of long-term behavior for each class.

**Figure 4:**
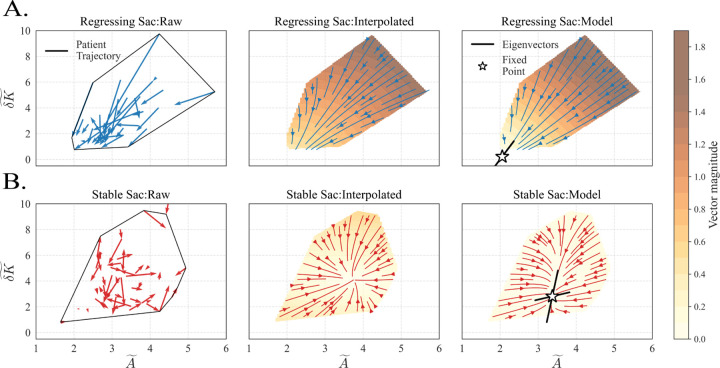
Cohort-level flow fields in normalized size–shape space. Panels illustrate empirical and modeled dynamics of sac remodeling in the $\left(\tilde{A},\tilde{\delta K}\right)$ plane for regressing (**A**, top row, blue) and stable (**B**, bottom row, red) post-EVAR sacs. *Raw:* finite-difference derivatives from longitudinal trajectories (black outlines represent the convex hull of observed states; arrows are the per-patient vectors). *Interpolated:* smoothed flow fields obtained by gridding/interpolating pooled derivatives with background shading proportional to vector magnitude. *Model:* mean Z–SINDy vector fields with fixed points (stars) and associated eigenvectors (black lines) indicating local stability structure. Eigenvalues of the linearized flow at each fixed point set local contraction/expansion rates; characteristic time scales τ=1/|Reλ| are ~5.0 years along both eigen directions for the regressing model (stable node with two negative real parts), and ~17.5 and 51.6 years for the stable model, reflecting much slower and direction-dependent remodeling.

To relate model-level dynamics to cohort behavior, we rendered population flow fields in the $\left(\tilde{A},\tilde{\delta K}\right)$ plane (Fig. 4). The *Raw* panels plot finite-difference patient derivatives overlaid on the convex hull of observed states, showing individual progression vectors: regressing sacs flow down and left, while stable sacs circulate within a higher-$\tilde{\delta K}$ region with comparatively small changes in $\tilde{A}$. *Interpolated* derivatives, projected onto the convex-hull grid, concentrate streamlines and motion magnitude, yielding average behavior for each cohort. The *Model* panels, generated by evaluating the mean Z–SINDy vector fields, mirror the empirical trends and demonstrate the effectiveness of simple parametric models. Stars mark fixed points predicted by each class-specific ODE; local eigenvectors (black lines) align with the principal directions of flow. The regressing model has a stable fixed point (both eigenvalues with negative real parts), whereas the stable model exhibits a saddle (eigenvalues with opposite signs). Characteristic time scales estimated from the eigenvalues of the linearized flow, via τ=1/|Reλ|, indicate substantial remodeling for regressing sacs (~5.0 years, both eigenvalues with similar negative real parts, consistent with a stable node) and much slower, anisotropic dynamics for stable sacs. The estimated time scales are consistent with the clinical observations: regressing sacs demonstrate substantial remodeling over the ~5 years of follow-up imaging, while stable sacs do not. Together, Figs. 3–4 show that (i) the two outcome groups are governed by distinct dynamics in a two-variable description and (ii) Z–SINDy reproduces cohort flow structure observed in the empirical trajectories while providing a unified system of equations for each outcome class. Throughout the Supplemental Information, we demonstrate that an affine function library is sufficient to capture the essential dynamics of both regressing and stable sacs. In particular, Fig. 8 compares Z–SINDy flow fields learned using second- and third-order polynomial libraries. No qualitative differences are observed: the regressing model continues to exhibit rapid descent toward nonpathological size–shape states, while the stable model retains a saddle structure at intermediate values of $\left(\tilde{A},\tilde{\delta K}\right)$. Higher-order terms introduce modest local curvature in the vector field but do not alter mean behavior. These results indicate that the observed cohort dynamics are low-order and structurally robust, supporting the use of minimal parametric models without loss of explanatory power.

### Bayesian classification and model stress tests

**Figure 5:**
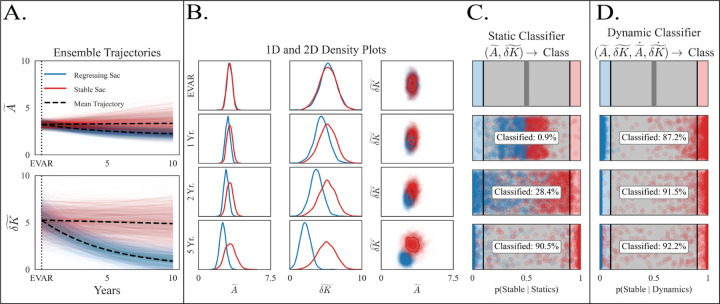
From Z–SINDy ensembles to static and dynamic Bayesian classification. **A** Ensembles of 10,000 simulated trajectories from the regressing (blue) and stable (red) Z–SINDy models for $\tilde{A}(t)$ (top) and $\tilde{\delta K}(t)$ (bottom). **B** Distributional views of the same ensembles: 1D marginals (left and middle) and joint densities (right) at EVAR, 1 yr, 2 yr, and 5 yr. The middle column and the inputs to panel C use identical point clouds. **C** Posterior probabilities from the static Bayesian classifier, pooling $p\left(x_{k}|c,t_{k}\right)$ over observed states. **D** Posterior probabilities from the dynamic Bayesian classifier using empirical derivatives $p\left({\dot{x}}_{k}|c\right)$. Under the dynamic classifier, patients exits the “uncertain” region earlier than the static classifier, reflecting the added value of directional information. Importantly, the inputs to C and D use the same underlying point clouds shown in B, enabling a direct comparison between raw ensemble separation and the decision structure of the static classifier.

Having established class-specific dynamics, we next asked whether the Z–SINDy models could support individualized, probabilistic classification of postoperative outcome and how early such decisions can be made under simulated real-world sampling. Figure 5A shows ensembles of 10,000 simulated trajectories generated from the two Z–SINDy models. These ensembles reflect plausible temporal evolutions under coefficient uncertainty for regressing (blue) and stable (red) sacs and already illustrate divergence over time.

Figure 5B reformats these ensembles into one- and two-dimensional distributional views across clinically meaningful reference times. Kernel-density estimates of $\tilde{A}$ and $\tilde{\delta K}$ marginals (left and middle) and their joint densities (right) summarize how the classes separate. Immediately after repair the two classes overlap; by two to five years their marginals and joint densities diverge substantially, with regressing sacs shifting toward lower size and shape while stable sacs maintain higher values.

Figure 5C applies the static Bayesian classifier, which evaluates the probability of being a stable sac, p(Stable|Statics), using only instantaneous coordinates in size–shape space. By contrast, Fig. 5D shows the dynamic Bayesian classifier, which evaluates likelihoods, p(Stable|Dynamics), thereby exploiting directional flow information rather than position alone. By convention, p=1 predicts a stable sac, while p=0 predicts a regressing sac. Across time points, the dynamic classifier produces higher-confidence predictions earlier: by one to two years post-EVAR, a larger fraction of patients exit the “uncertain” band (0.1<p<0.9) in Fig. 5D than in Fig. 5C. This behavior is reproduced in Supplementary Fig. 11, where the dynamic classifier consistently concentrates posterior mass toward the correct class earlier than the static classifier, using both CT-only data and CT data augmented with FEA-based upsampling, and remains robust under K-fold cross-validation (Table 1).

**Figure 6:**
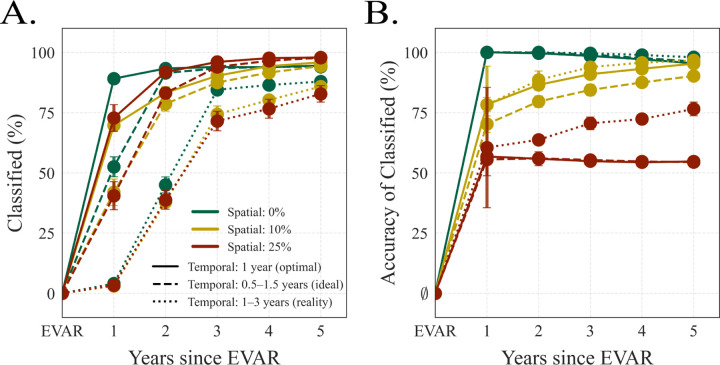
Robustness of the dynamic Bayesian classifier to spatial and temporal noise. **A:** fraction of scans confidently classified $\left({\text{max}}_{c}p(c|\cdot )\geq 0.9\right)$ versus time since EVAR. **B:** accuracy among the classified subset. Colors encode spatial noise added independently to $\tilde{A}$ and $\tilde{\delta K}$ prior to classification (green: none; yellow: 10% of feature magnitude; red: 25%). Line styles encode temporal sampling: solid = exactly annual (*Optimal 1.0*); dashed = jittered intervals randomly sampled from [0.5, 1.5] yr (*Ideal*); dotted = sparse/irregular intervals from [1.0, 3.0] yr (*Reality*). The EVAR baseline corresponds to the non-informative 50% prior with essentially no scans classified. Error bars represent variability across 50 realizations. The classifier confidently labels an increasing fraction of scans over time, with accuracy remaining high except under the most severe combination of spatial noise and temporal sparsity.

Finally, we quantified the robustness of classification to clinically realistic spatial and temporal noise (Fig. 6). We injected controlled spatial noise into $\left(\tilde{A},\tilde{\delta K}\right)$ and coarsened sampling from exactly annual (*Optimal 1.0*) to jittered (*Ideal*, 0.5–1.5 yr) and sparse/irregular (*Reality*, 1–3 yr) intervals, re-estimating derivatives and reapplying the dynamic classifier. The left panel shows the fraction of scans that meet the high-confidence threshold $\left({\text{max}}_{c}p(c|\cdot )\geq 0.9\right)$; the right panel shows the accuracy among this classified subset. Three consistent trends emerged. (i) The proportion of confidently classified scans is systematically reduced by temporal irregularity rather than spatial noise. (ii) When spatial noise is modest (0–10%), accuracy for the classified subset remains high (≳ 90%) even under non-ideal sampling, indicating that most confident decisions are correct. (iii) Under the most aggressive corruption (25% spatial noise with sparse/irregular sampling), accuracy drifts toward the non-informative prior and highlighting the regime in which measurement error and follow-up gaps overwhelm the dynamical signal. Supplementary Fig. 13 further extends these stress tests by incorporating real-world patient attrition (Fig. 12) and time-dependent class priors (Supplementary Section S8). Under these clinically realistic conditions, the dynamic classifier again exits the uncertain region earlier than the static classifier (well before the majority of patients are lost to follow-up), reinforcing the translational importance of modeling aortic remodeling as a dynamic system rather than static process.

## Discussion

This work leverages a physically interpretable, anatomically grounded approach to model postoperative AAA remodeling and uses the fitted models for clinically actionable classification. Motivated by the limitations of size-only assessments and by sparse, irregular follow-up, we framed sac regression or stabilization as dynamics in a low-dimensional, interpretable state-space described by normalized size $\left(\tilde{A}\right)$ and shape $\left(\tilde{\delta K}\right)$. Two main findings emerged: (i) outcome-specific Z–SINDy models capture cohort dynamics and yield meaningful fixed points (Figs. 3–4); and (ii) these dynamics enable probabilistic classification that exploits dynamical directional information rather than position alone in size–shape space, providing earlier and more accurate decisions that can be stress-tested for robustness (Figs. 5–6).

### Dynamical forecasting

A central challenge for clinically useful time-series models is balancing accuracy with interpretability. By restricting the library to linear terms, Z–SINDy identifies the simplest models consistent with the data while preserving a direct physiological mapping between features and coefficients [32]. For regressing sacs, trajectories trend toward a stable fixed point near $\left(\tilde{A},\tilde{\delta K})\approx [1,1\right]$, i.e., the definition of a nonpathological aorta in the normalized space; the characteristic time scale (~5 years) matches typical follow-up imaging. For stable sacs, the mean field exhibits a fixed point with much longer time scales; the model predicts near-constant $\tilde{A}$ with persistently higher $\tilde{\delta K}$ on clinical time horizons. Importantly, Fig. 4 closes the loop between model and data: raw finite-difference vectors, interpolated flow fields, and Z–SINDy vector fields show consistent directions across the observed convex hull, indicating that the learned ODEs reflect underlying cohort behavior.

### Bayesian classifiers

Translating learned dynamics into clinical utility requires principled decision-making under measurement uncertainty. Our first practical contribution is a pair of Bayesian classifiers—*static* (coordinate-based) and *dynamic* (derivative-based)—that take an individual patient’s trajectory and return the posterior probability of regressing or stable outcome. Because derivatives encode *where a patient is heading* rather than *where they are now*, the dynamic classifier becomes discriminative sooner: by 1 year it confidently classifies 87% of patients with high predictive accuracy, whereas the static classifier only classifies 1% of patients (Fig. 5). This is crucial in practice, where only 2–3 scans may be available per patient and early risk stratification can guide surveillance and reintervention strategy. While static or instantaneous characterization of aortic morphology remains useful, dynamic information of anatomic trajectory is invaluable. Clinically, we need to identify non-regressing AAA sacs at one year post-EVAR, not wait until they don’t follow up at year 5. The Society for Vascular Surgery’s (SVS) Vascular Quality Initiative (VQI) shows loss-to-follow-up is associated with a 6.45-fold higher mortality risk underscoring the need for early risk identification and proactive surveillance [33]. We’ve seen similar trends with aortic dissection patients at our own institution [34]. Supplementary analyses (Supplemental Section S8) of different attrition rates of regressing and stable cohorts indicate that the mere existence of a patient’s follow-up CT scan several years post-EVAR can enhance the classification regardless of the specific scan outcome.

### Stress testing and study design

Our second contribution is explicit quantification of how spatial measurement error (e.g., CT resolution and segmentation variability) [35, 36, 37] and temporal irregularity (loss to follow-up, variable scheduling) [38, 39, 40, 41] influence performance of the classifier (Fig. 6). Three conclusions follow. (i) Accuracy improves monotonically with postoperative time across conditions, reflecting accumulating information and growing separation between outcomes. (ii) Spatial noise degrades performance in a predictable fashion; when perturbations reach roughly a quarter of the feature scale, performance approaches the 50% prior. (iii) Temporal irregularity induces meaningful losses relative to exactly annual imaging, emphasizing the value of timely scans for derivative estimation. These stress tests provide a quantitative lens for planning studies, enabling investigators to trade off sample size, cadence, and anticipated noise to meet prespecified accuracy targets.

The proposed framework suggests an anatomically defined, rate-based approach to post-EVAR surveillance. The size–shape mapping is straightforward to compute from standard CT-derived segmentations, and Z–SINDy has an adaptable open-source implementation [42]. Posterior-predictive simulations generate reference distributions for both outcomes, supporting downstream analyses and visualization. The framework naturally accommodates new data as additional scans become available, enabling adaptive surveillance: patients whose dynamic posteriors favor the regressing model might maintain standard follow-up, whereas those with persistently high $\tilde{\delta K}$ could be flagged for closer evaluation or adjunctive treatment. More broadly, summarizing cohort behavior with fixed points (and eigenvectors) aids communication by distilling complex remodeling into intuitive descriptors (“which fixed point are you approaching, and how fast?”).

### Future work

Our formulation rests on simplifying geometric, dynamical, and clinical assumptions that guide future extensions. *Geometry:* we deliberately restricted the feature space to $\tilde{A}$ and $\tilde{\delta K}$ to highlight size–shape coupling; richer variables (e.g., thrombus morphology, graft type, hemodynamics) may refine subgroup structure. The fluctuation in integrated Gaussian curvature δK is empirically informative [10] but remains a heuristic; principled shape metrics from differential geometry could be incorporated [43]. *Dynamics:* we used a linear library to balance fit and interpretability; given current data volume/noise, nonlinear extensions offer limited gains, but future datasets may warrant higher-order terms. Measurement noise chiefly affects empirical derivatives $\left(\dot{\tilde{A}},\dot{\tilde{\delta K}}\right)$; adaptive denoising and derivative-regularization methods could further stabilize estimates in clinical settings [44, 45, 46, 47]. *Clinical scope:* here we modeled two classes within AAA. Extending to expanding sacs (Type I endoleak or pre-rupture), thoracic aneurysms, post-TEVAR remodeling, and other morphology-driven conditions—paired with stress-test paradigms to inform surveillance design and quality standards—is a natural trajectory for further research. Heterogeneity across devices, imaging practices, and segmentation quality was embraced rather than harmonized, underscoring the need for prospective, multi-institutional validation of dynamical modeling approaches.

By reframing AAA remodeling as inference of simple, physically meaningful dynamics, we bridge mechanistic understanding and clinical decision-making. Z–SINDy provides compact models that match cohort behavior and yield interpretable flow structure; Bayesian classifiers convert those models into early, patient-specific probabilities; and stress tests reveal when and how performance degrades under real-world constraints. Together, these elements support translational progress toward reliable, transparent, and resource-aware modeling that can inform how, when, and for whom postoperative surveillance should adapt.

## Methods

### Clinical data and morphological feature space

We curated longitudinal CT angiography for EVAR patients, each contributing 2–5 usable time points spanning months to years. From each segmented sac we computed the sac surface area A and a shape descriptor defined as the fluctuation in integrated Gaussian curvature, δK. After scale averaging, both were normalized by cohort means to yield dimensionless features $\tilde{A}$ and $\tilde{\delta K}$; the patient state is $x(t)=[\tilde{A}(t),\tilde{\delta K}(t){]}^{⊤}$. (Operational details of scale averaging and curvature computation follow our prior work [48].)

### Temporal resampling and derivative estimates

To mitigate irregular follow-up, each subject’s time series was upsampled to a quasi-regular grid and finite-difference derivatives were computed on the resampled series. We enforced a minimum separation between scans used for differencing and removed clearly non-physiologic spikes in d $\tilde{\delta K}/\text{d}t$ (Supplementary Sections S1–S2).

### Sparse identification of dynamics (Z–SINDy)

We modeled the temporal evolution of x(t) using Z–SINDy [30]. In the main text we use an affine library

$$\Theta (x)=[1,\tilde{A},\tilde{\delta K}{]}^{⊤},\;\dot{x}\left(t\right)\approx \Xi^{⊤}\Theta \left(x\left(t\right)\right),$$

with Bayesian estimation of coefficients Ξ and a data-matched resolution parameter ρ obtained from residual variance. Model uncertainty enters through the posterior over Ξ, which we use to generate trajectory ensembles and error bars. The complete likelihood/prior specification, posterior formulas, and hyperparameter sweep are provided in Supplementary Section S3.

### Flow fields, fixed points, and uncertainty visualization

We compared empirical finite-difference vectors with model-predicted dynamics in the $\left(\tilde{A},\tilde{\delta K}\right)$ plane by rendering (i) raw arrows at scan locations, (ii) an interpolated cohort field over the empirical convex hull, and (iii) the mean Z–SINDy vector field with streamline seeding proportional to data density. Fixed points of the mean ODEs were obtained by solving $\dot{x}(x)=0$; local eigenvectors are plotted to indicate stability structure. Forecast bands reflect coefficient-posterior sampling (Supplementary Sections S4–S5).

### Bayesian classification

We built two complementary classifiers: (i) a dynamic classifier that evaluates the likelihood of observed derivatives under each class-specific Z–SINDy model, and (ii) a static classifier that evaluates the likelihood of observed states against time-indexed simulated ensembles. In all analyses we used equal class priors p(c)=1/2 and declared a confident decision when ${\text{max}}_{c}p(c|\cdot )\geq 0.9$; the corresponding likelihoods and normalization are detailed in Supplementary Section S6.

### Robustness to clinical sparsity: noise and sampling inhomogeneity stress tests

To emulate real-world data degradation, we applied controlled corruptions to the resampled trajectories before classification. First, we injected spatial Gaussian noise with a standard deviation set to a fixed percentage of each feature’s magnitude (e.g., 10% and 25% of $\tilde{A}$ and $\tilde{\delta K}$), independently at each time point. Second, we used either regular temporal sampling (e.g., annual intervals) or random time increments within bounds (e.g., 0.5–1.5 years), thereby reducing effective time resolution to stress the dynamic classifier. After corruption, we re-estimated derivatives where applicable, reapplied the dynamic classifier, and quantified shifts in posterior class probabilities, directly assessing sensitivity to segmentation/measurement noise and to attrition typical of clinical datasets.

## Supplementary Material

1
