# Supplementary material for: Dynamic Temporal Modeling of Abdominal Aortic Aneurysm Morphology with Z–SINDy
Source: medRxiv. 2026 Jan 5:2025.09.29.25336910. Preprint. [Version 2] doi: 10.1101/2025.09.29.25336910 (PMC12803291; doi:10.1101/2025.09.29.25336910)
Supplement: 1 [file NIHPP2025.09.29.25336910V2-supplement-1.pdf]

medRxiv preprint doi: https://doi.org/10.1101/2025.09.29.25336910; this version posted January 5, 2026. The copyright holder for this preprint (which was not certified by peer review) is the author/funder, who has granted medRxiv a license to display the preprint in perpetuity.
It is made available under a CC-BY 4.0 International license .

Supplemental Information

In addition to this additional information, the code used for data processing, model construction, and analysis is publicly available at https://github.com/SurgBioMech/aaa-dynamics. The anatomic feature data (per-patient, per-CT) supporting the findings of this study is available from the same repository.

S1. FEA-based up-sampling and polynomial resampling

To address irregular clinical follow-up and inherent sparsity, we upsampled trajectories using a two-step procedure. Using finite-element analysis (FEA) simulations, we first constructed dense surrogate trajectories for A and δK that respect sac mechanics (see prior work for details [31]). These sequences furnish physically plausible intermediate states between clinical scans without extrapolating beyond the postoperative ground truth $\mathbf{x}(t_f)$. We then resampled each subject's trajectory by fitting a third-order polynomial to each feature as a function of time and evaluating the fit at fixed increments of $\Delta t = 0.26$ and 0.14 years. These increments were chosen by taking the mean inter-scan intervals in the regressing (2.6 yr) and stable (1.4 yr) datasets and dividing each by 10. If this procedure generated more points than a preset per-subject limit, we instead produced exactly $N = 30$ evenly spaced samples between the first and last observed scan. This soft cap prevents oversampling of long trajectories while preserving short-timescale variation. The resulting dataset is sufficiently upsampled and evenly spaced for stable derivative estimation and dynamical modeling, while mitigating variance stemming from irregular clinical follow-up and patient attrition.

S2. Derivative estimation and outlier handling

We estimated time derivatives using pairwise finite differences between successive observations within subject. To avoid spuriously large derivatives from nearly coincident scans, we required a minimum time separation of 3 months. Let $(t_i, \widetilde{A}_i, \widetilde{\delta K}_i)_{i=1}^{N}$ denote the ordered time points and corresponding normalized features for a subject. To estimate instantaneous rates of change $(\dot{\widetilde{A}}, \dot{\widetilde{\delta K}})$, we applied second-order central differences to the compressed time

medRxiv preprint doi: https://doi.org/10.1101/2025.09.29.25336910; this version posted January 5, 2026. The copyright holder for this preprint (which was not certified by peer review) is the author/funder, who has granted medRxiv a license to display the preprint in perpetuity.
It is made available under a CC-BY 4.0 International license .

series:

$$\dot{\widetilde{A}}_i = \frac{\widetilde{A}_{i+1} - \widetilde{A}_{i-1}}{t_{i+1} - t_{i-1}}, \quad \dot{\widetilde{\delta K}}_i = \frac{\widetilde{\delta K}_{i+1} - \widetilde{\delta K}_{i-1}}{t_{i+1} - t_{i-1}}, \quad \text{for } i = 2, \ldots, N-1. \tag{1}$$

We also applied light, physically motivated outlier rejection on $\mathrm{d}\,\widetilde{\delta K}/\mathrm{d}t$ rates to remove biologically implausible spikes introduced by algorithmic error (segmentation, curvature estimation, or timestamps). Specifically, if

$$\frac{\mathrm{d}\,\widetilde{\delta K}}{\mathrm{d}t} > 6 \text{ yr}^{-1} \quad \text{or} \quad < -3 \text{ yr}^{-1},$$

the offending scans were removed from the dataset, and the remaining data were then used to fit the dynamical model.

S3. Z–SINDy inference details, hyperparameters, and linear-library justification

We model the temporal evolution of the state $\mathbf{x}(t) = [\widetilde{A}(t), \widetilde{\delta K}(t)]^\top$ as $\dot{\mathbf{x}}(t) \approx \mathbf{\Xi}^\top \mathbf{\Theta}(\mathbf{x}(t))$, where in the main text we restrict the library to the affine form $\mathbf{\Theta}(\mathbf{x}) = [1, \widetilde{A}, \widetilde{\delta K}]^\top$ (alternative higher-order libraries are explored here for completeness). Parameters $\mathbf{\Xi}$ are inferred in a Bayesian framework with a Gaussian likelihood that penalizes the squared residuals between observed derivatives and model predictions,

$$p(\{\dot{\mathbf{x}}\} \mid \mathbf{\Xi}) \propto \prod_l \exp\left[-\frac{1}{2\rho^2} \sum_k \left(\dot{x}_{l,k} - \mathbf{\Xi}_l^\top \mathbf{\Theta}(\mathbf{x}_k)\right)^2\right], \tag{2}$$

and a sparsity-inducing Bernoulli–Gaussian prior over active-set indicators γ, $p(\mathbf{\Xi}_l) \propto \prod_i \left(\delta(\Xi_{l,i}) + w(\Xi_{l,i})\, e^{-\lambda n}\right)$, with λ controlling parsimony. Conditioning on a fixed active set γ yields a multivariate Gaussian posterior for the coefficients,

$$\mathbf{\Xi}_l \mid \{\dot{\mathbf{x}}\} \sim \mathcal{N}(\boldsymbol{\mu}_l, \mathbf{\Sigma}), \qquad \boldsymbol{\mu} = \mathbf{C}^{-1}\mathbf{V}, \quad \mathbf{\Sigma} = \rho^2\, \mathbf{C}^{-1}, \tag{3}$$

where $\mathbf{C} = \sum_k \mathbf{\Theta}(\mathbf{x}_k)\mathbf{\Theta}(\mathbf{x}_k)^\top$ and $\mathbf{V} = \sum_k \mathbf{\Theta}(\mathbf{x}_k)\dot{\mathbf{x}}_k^\top$; the full posterior is a Gaussian mixture $\sum_\gamma p(\mathbf{\Xi} \mid \gamma)\, \mathcal{Z}_\gamma$ typically dominated by a single γ. We estimate the resolution parameter from residual variance, $\rho \approx \sqrt{(dn)^{-1} \sum_{k,l} (\dot{x}_{l,k} - \boldsymbol{\mu}_l^\top \mathbf{\Theta}(\mathbf{x}_k))^2}$, and, where relevant, sweep the regularization weight λ to balance parsimony and fit by explained variance. This inference supports uncertainty propagation by sampling $\mathbf{\Xi}^{(m)} \sim \mathcal{N}(\boldsymbol{\mu}, \mathbf{\Sigma})$ to generate trajectory ensembles and confidence bands, and by analyzing fixed points/eigenstructure of the mean model (procedures detailed in Supplementary Methods S4–S5). Crucially, the hyperparameter sweep in Fig. 7 shows that the linear (affine) library already captures the bulk of explainable variance for both cohorts across the central range of λ, whereas quadratic/cubic libraries offer negligible gains and may reduce R^2 at extreme sparsity—motivating our Occam-optimal choice of a linear library in the main analyses. Fig. 8 demonstrates the vector fields of higher-order models that reproduce the key dynamical features of the linear models. Complementing this fit–complexity tradeoff, Fig. 9 demonstrates coefficient reproducibility under randomized train splits: split-wise means concentrate near the across-split mean with narrow one–standard–deviation bars relative to coefficient magnitudes. Together,

medRxiv preprint doi: https://doi.org/10.1101/2025.09.29.25336910; this version posted January 5, 2026. The copyright holder for this preprint (which was not certified by peer review) is the author/funder, who has granted medRxiv a license to display the preprint in perpetuity.
It is made available under a CC-BY 4.0 International license .

Figs. 7–9 justify restricting $\boldsymbol{\Theta}$ to linear terms and indicate that the inferred dynamical functions are well constrained by the data and robust to moderate perturbations of the training set.

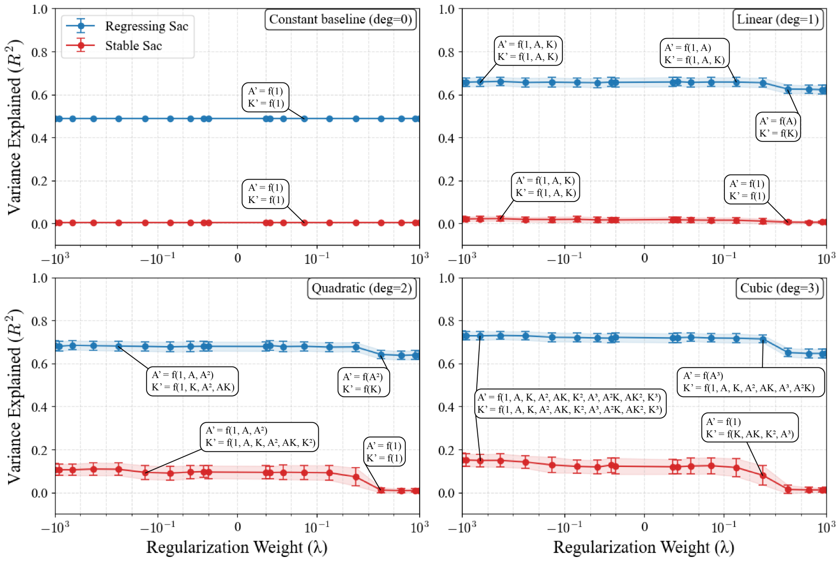

Figure 7: Hyperparameter sweep for Z–SINDy model fit. Mean variance explained (R^2) ± 1 SD versus regularization weight λ for four polynomial libraries of increasing maximal degree: constant (deg$=$ 0), linear (deg$=$ 1), quadratic (deg$=$ 2), and cubic (deg$=$ 3). Blue curves: regressing sac cohort; red curves: stable sac cohort. For each degree and λ, Z–SINDy is fit to $(\widetilde{A}, \widetilde{\delta K})$ and their time derivatives using the cohort-specific resolution parameter ρ. Uncertainty bands are obtained by sampling from the coefficient covariance of the Gaussian posterior. The x-axis uses a logarithmic scale to show the hyperparameter axis about $\lambda = 0$. Call outs denote representative active term sets in the learned right-hand sides (e.g., $d\widetilde{A}/dt = f(1, \widetilde{A}, \widetilde{\delta K})$). Relative to the constant baseline, linear models already capture most explainable variance for the regressing cohort, while higher-order terms tend to overfit the derivative training data.

medRxiv preprint doi: https://doi.org/10.1101/2025.09.29.25336910; this version posted January 5, 2026. The copyright holder for this preprint (which was not certified by peer review) is the author/funder, who has granted medRxiv a license to display the preprint in perpetuity.
It is made available under a CC-BY 4.0 International license .

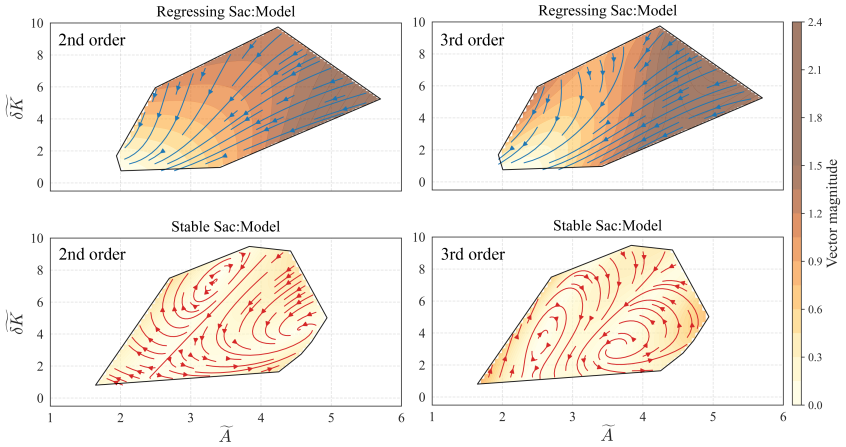

Figure 8: Z–SINDy vector fields with higher-order polynomial libraries. Mean cohort-level flow fields in the normalized size–shape space ($\widetilde{A}$, $\widetilde{\delta K}$) for regressing (top row) and stable (bottom row) sacs, analogous to the *Model* panels of Fig. 4 but using polynomial libraries up to second- and third-order. Left column: quadratic library (up to 2nd order); right column: cubic extension (up to 3rd order). Streamlines indicate characteristic trajectories and background shading encodes vector magnitude. Increasing the polynomial degree modestly warps streamlines and local speeds but preserves the qualitative flow structure observed for the affine (main-text) models—faster convergence toward a low-size/low-shape region for regressing sacs and slower recirculation within a higher-$\widetilde{\delta K}$ band for stable sacs—demonstrating that the main dynamical conclusions are robust to reasonable expansions of the function library.

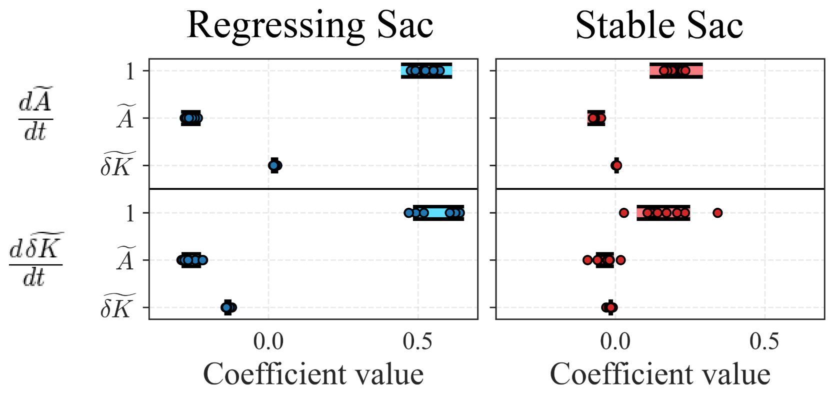

Figure 9: Coefficient distributions for the affine Z–SINDy model. Aggregated coefficient summaries for the linear (affine) library $\Theta(x) = [1, \widetilde{A}, \widetilde{\delta K}]$ in each differential equation (top: $d\widetilde{A}/dt$; bottom: $d\widetilde{\delta K}/dt$). Left panel: regressing sac model (blue); right panel: stable sac model (red). The dataset is repeatedly subsampled into randomized train splits; the model is re-fit on each split; and the resulting coefficients are summarized at the term level. Small circles show split-wise means; thick colored horizontal bars show mean $\pm$ one standard deviation across splits, overlaid on a thin black bar marking the same interval. This compact, uncertainty-aware view reveals which contributions (constant, $\widetilde{A}$, $\widetilde{\delta K}$) are reproducibly nonzero in each equation and how their magnitudes differ between cohorts.

medRxiv preprint doi: https://doi.org/10.1101/2025.09.29.25336910; this version posted January 5, 2026. The copyright holder for this preprint (which was not certified by peer review) is the author/funder, who has granted medRxiv a license to display the preprint in perpetuity.
It is made available under a CC-BY 4.0 International license .

S4. Uncertainty-aware simulation and fixed-point analysis

We quantified forecast uncertainty by generating ensembles via sampling model coefficients from the posterior $\mathbf{\Xi}^{(m)} \sim \mathcal{N}(\boldsymbol{\mu}, \mathbf{\Sigma})$ and initial conditions from a Gaussian around a preoperative baseline. Each sampled model was integrated forward to obtain trajectories $\mathbf{x}^{(m)}(t)$. We visualized uncertainty with shaded bands about the mean trajectory for each class (*regressing* and *stable*) for $\mathbf{x}(t)$ and for $\dot{\mathbf{x}}(t) = \mathbf{\Xi}^{(m)\top}\mathbf{\Theta}(\mathbf{x}(t))$. Fixed points $\mathbf{x}^*$ were computed from the mean model by solving $\dot{\mathbf{x}}(\mathbf{x}) = 0$ and are plotted as horizontal lines in the respective panels.

S5. Population flow-field visualization in morphological space

To compare empirical patient trajectories with model-predicted dynamics, we visualized cohort-level flow fields in the $(\widetilde{A}, \widetilde{\delta K})$ plane. For the original CT-derived data (labeled *Raw*), we computed the convex hull of all observed states and overlaid finite-difference estimates of $\left(\mathrm{d}\,\widetilde{A}/\mathrm{d}t,\ \mathrm{d}\,\widetilde{\delta K}/\mathrm{d}t\right)$ as arrows anchored at the corresponding scan locations. Next, these derivative estimates were pooled across patients and interpolated onto a regular grid spanning the convex hull, with the background magnitude $\|\dot{\mathbf{x}}\|$ rendered to indicate local dynamic speed (labeled *Interpolated*). The interpolation was performed with radial basis-functions (RBF) with the smoothing parameter tuned to the plateau of variance explained (Fig. 10). Streamlines were seeded using a kernel-density estimate over the empirical point cloud so that streamline density reflects regions with data support. Lastly, we evaluated the mean Z–SINDy vector field for each class, rendering streamlines and shading by vector magnitude (labeled *Model*). Fixed points from the mean ODEs are marked with stars, and associated eigenvectors are shown as black lines to indicate local stability structure.

medRxiv preprint doi: https://doi.org/10.1101/2025.09.29.25336910; this version posted January 5, 2026. The copyright holder for this preprint (which was not certified by peer review) is the author/funder, who has granted medRxiv a license to display the preprint in perpetuity.
It is made available under a CC-BY 4.0 International license .

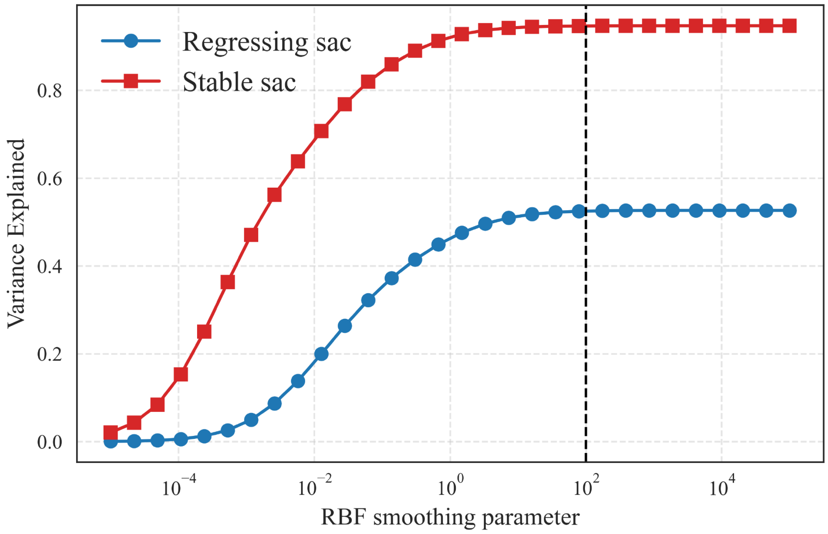

Figure 10: Tuning the smoothing scale for empirical vector fields. Normalized root-mean-square error (RMSE) between the radial basis–function (RBF) smoothed flow field and the raw finite-difference derivatives as a function of the RBF smoothing parameter for the regressing (blue circles) and stable (red squares) cohorts. Errors are normalized so that values near zero indicate close agreement with the unsmoothed empirical vectors. As smoothing increases, the RMSE rises and then plateaus; the dashed vertical line at 10^2 marks the smoothing parameter used for the interpolated flow fields in Fig. 4. Choosing this value lies at the onset of the plateau, providing visual regularization of the empirical field without appreciably degrading fidelity to the original derivative data.

S6. Bayesian classifiers: full likelihoods and normalization

The Bayesian classifiers take per-patient observations and convert them into posterior probabilities for the two outcome classes, $c \in \{\text{regressing}, \text{stable}\}$. Throughout this section we denote the state by $\mathbf{x}_k = [\widetilde{A}_k, \widetilde{\delta K}_k]^\top$ at time t_k and, where available, its finite-difference derivative $\dot{\mathbf{x}}_k$. Class-specific Z–SINDy models are summarized by coefficient matrices $\mathbf{\Xi}_c$ and resolution parameters ρ_c (Sec. S3). Unless otherwise noted, we use equal class priors $p(c) = 1/2$; attrition-aware variants with time-dependent priors are described in Sec. S8.

Dynamic classifier. The dynamic classifier evaluates how likely the observed derivatives $\{\dot{\mathbf{x}}_k\}$ are under each class-specific Z–SINDy model. Conditioning on class c, we assume independent Gaussian errors with variance ρ_c^2 about the model-predicted derivatives $\mathbf{\Xi}_c^\top \mathbf{\Theta}(\mathbf{x}_k)$, where $\mathbf{\Theta}$ is the chosen library:

$$z_c = p(\{\dot{\mathbf{x}}_k\} \mid c) = \prod_{k,\ell} \frac{1}{(2\pi\rho_c^2)^{1/2}} \exp\left[-\frac{1}{2\rho_c^2}\left(\dot{x}_{k,\ell} - \mathbf{\Xi}_{c,\ell}^\top \mathbf{\Theta}(\mathbf{x}_k)\right)^2\right].$$

Bayes' rule then yields the posterior class probability

$$p(c \mid \{\dot{\mathbf{x}}_k\}) = \frac{z_c\, p(c)}{\sum_{c'} z_{c'}\, p(c')}, \qquad p(c) = \tfrac{1}{2}.$$

medRxiv preprint doi: https://doi.org/10.1101/2025.09.29.25336910; this version posted January 5, 2026. The copyright holder for this preprint (which was not certified by peer review) is the author/funder, who has granted medRxiv a license to display the preprint in perpetuity.
It is made available under a CC-BY 4.0 International license .

In words, the dynamic classifier compares how well each class-specific ODE explains the observed motion in size–shape space; patients are assigned to the class whose model best predicts their derivatives.

Static classifier. The static classifier, by contrast, operates on coordinates rather than derivatives. For each class c we generate a large ensemble of trajectories by sampling initial conditions and coefficients from the class-specific Z–SINDy posterior and integrating forward in time (Sec. S4). These ensembles are used to estimate time-indexed densities $p(\mathbf{x} \mid c, t)$ over the state space. Given a patient's observations $\{(\mathbf{x}_k, t_k)\}$, we assume conditional independence across time and compute the class likelihood

$$z_c = \prod_k p(\mathbf{x}_k \mid c, t_k), \qquad p(c \mid \{\mathbf{x}_k, t_k\}) = \frac{z_c\, p(c)}{\sum_{c'} z_{c'}\, p(c')}.$$

The static classifier therefore reflects where a patient is relative to the simulated class ensembles at the same follow-up times, whereas the dynamic classifier reflects where the patient is according to the class-specific ODEs.

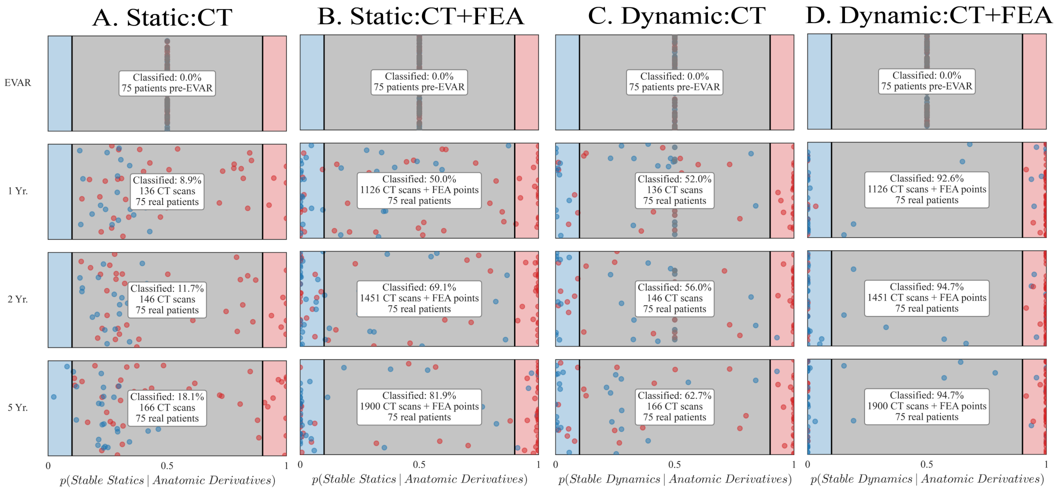

Figure 11: Posterior probability jitter plots for static and dynamic Bayesian classifiers across both real patient CT scans and synthetic data from the Z–SINDy models. Columns show (from left to right): A. Static classifier with CT data, B. Static classifier with synthetic data, C. Dynamic classifier with CT data, and D. Dynamic classifier with synthetic data. Rows correspond to EVAR (preoperative baseline) and 1, 2, and 5 years post-EVAR. The horizontal axis in each panel is the posterior probability of the stable class, either $p(\text{Stable Statics} \mid \text{anatomic derivatives})$ or $p(\text{Stable Dynamics} \mid \text{anatomic derivatives})$; blue and red points represent regressing and stable sacs, respectively. Vertical side bands at $p = [0.1, 0.9]$ indicate decision regions for each class, and the central grey band marks the "uncertain" region in which neither class exceeds the confidence threshold. In-panel boxes report the percentage of scans confidently classified, together with the number of scans and real or synthetic patients contributing to each panel.

Decision rule and performance metrics. For both classifiers we declare a confident decision when the posterior for one class exceeds a fixed probability threshold, $\max_c p(c \mid \cdot) \geq 0.9$; otherwise the scan is labeled "uncertain" and excluded from accuracy calculations.

medRxiv preprint doi: https://doi.org/10.1101/2025.09.29.25336910; this version posted January 5, 2026. The copyright holder for this preprint (which was not certified by peer review) is the author/funder, who has granted medRxiv a license to display the preprint in perpetuity.
It is made available under a CC-BY 4.0 International license .

The fraction of scans meeting this criterion is reported as "Classified (%)", and the accuracy is computed over this subset as the percentage whose predicted class matches the clinical label. Figure 11 shows the distribution of posterior probabilities for each classifier and dataset (CT only versus the FEA upsampled datasets), with the central grey band marking the uncertain region $0.1 < p(\text{Stable} \mid \cdot) < 0.9$. The cross-validated summary in Table 1 quantifies these patterns: the dynamic classifier confidently labels a much larger fraction of CT-only scans at early times than the static classifier, and the introduction of upsampled data from FEA simulations further increases both the fraction classified and the resulting accuracy, especially at 1–2 years post-EVAR.

Table 1: Cross-validated performance of dynamic and static Bayesian classifiers. Within each of five folds, Z–SINDy models are trained on random 80% of patients and classification is applied to the remaining 20%. Entries are mean±std across folds.

Classifier	Dataset	Time (yr)	Classified (%)	Accuracy (%)
Static	CT	1	10 ± 10	100 ± 0
		2	12 ± 9	100 ± 0
		5	19 ± 17	100 ± 0
	CT+FEA	1	50 ± 6	77 ± 18
		2	69 ± 13	74 ± 16
		5	82 ± 7	76 ± 11
Dynamic	CT	1	52 ± 12	88 ± 13
		2	56 ± 12	82 ± 13
		5	63 ± 11	83 ± 12
	CT+FEA	1	93 ± 5	80 ± 14
		2	95 ± 4	81 ± 13
		5	95 ± 6	82 ± 12

S7. Stress-test protocols (noise and sampling inhomogeneity)

We evaluate robustness by corrupting the resampled series prior to classification: (i) add independent Gaussian noise at each time point with SD equal to a fixed fraction of each feature's magnitude (e.g., 10%, 25% for $\widetilde{A}$, $\widetilde{\delta K}$); (ii) impose either regular sampling (e.g., annual) or random increments in $[0.5, 1.5]$ years to reduce temporal resolution. We then recompute derivatives (for the dynamic classifier), re-estimate posteriors, and quantify probability shifts.

S8. Attrition-aware Bayesian classification

To quantify how loss to follow-up influences outcome prediction, we incorporated the empirically observed patient attrition rates for each clinical cohort into both the static and dynamic Bayesian classifiers. Fig. 12 summarizes the declining proportions of stable and regressing patients remaining at each postoperative time point following EVAR. For each class, the Bayesian classifiers evaluated likelihoods using time-dependent priors equal to the linearly interpolated fraction of patients still under surveillance.

To emulate the availability of real clinical data, ensembles of simulated trajectories were additionally subjected to randomized dropout consistent with the same patient attrition

medRxiv preprint doi: https://doi.org/10.1101/2025.09.29.25336910; this version posted January 5, 2026. The copyright holder for this preprint (which was not certified by peer review) is the author/funder, who has granted medRxiv a license to display the preprint in perpetuity.
It is made available under a CC-BY 4.0 International license .

curves. Therefore, both the effective class priors and the number of trajectory realizations contributing to posterior estimates changed over time in a manner that reflects real-world surveillance behavior. Fig. 13 shows posterior probabilities for the attrition-aware classifiers. The dynamic classifier once again exits the "uncertain" region earlier than the static classifier. As shown in Fig. 5, at 5 years following EVAR (with no awareness of patient dropout), the static model classifies a similar percentage of trajectories compared to the dynamic model. However, the observed attrition rates show that by this time, there are less than 30% of the regressing cohort remaining and less than 5% of the stable cohort remaining. This highlights the significance of using morphologic dynamics to stratify patients at times earlier than static measures are able to inform clinical decision-making.

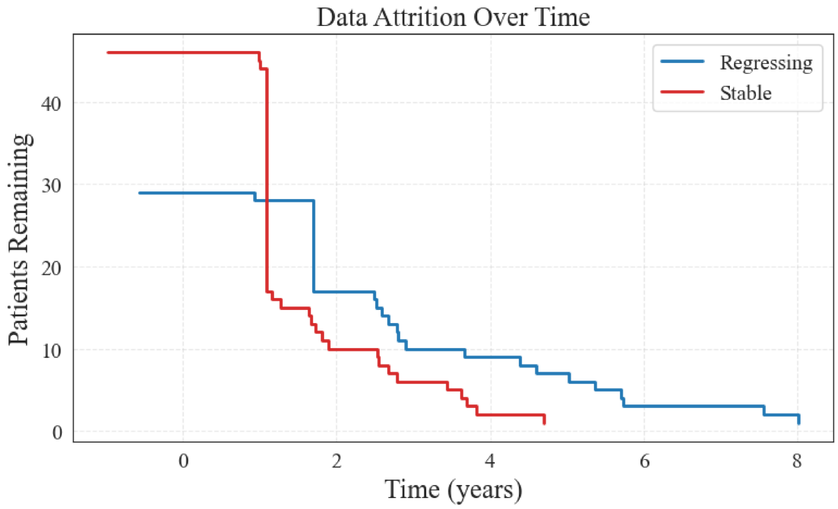

Figure 12: Quantities of regressing and stable patients in the time following EVAR (time=0). The differing rates of attrition for each class were incorporated into the stable and dynamic Bayesian classifiers, informing the models of clinically-observed patient distributions and loss to follow-up.

medRxiv preprint doi: https://doi.org/10.1101/2025.09.29.25336910; this version posted January 5, 2026. The copyright holder for this preprint (which was not certified by peer review) is the author/funder, who has granted medRxiv a license to display the preprint in perpetuity.
It is made available under a CC-BY 4.0 International license .

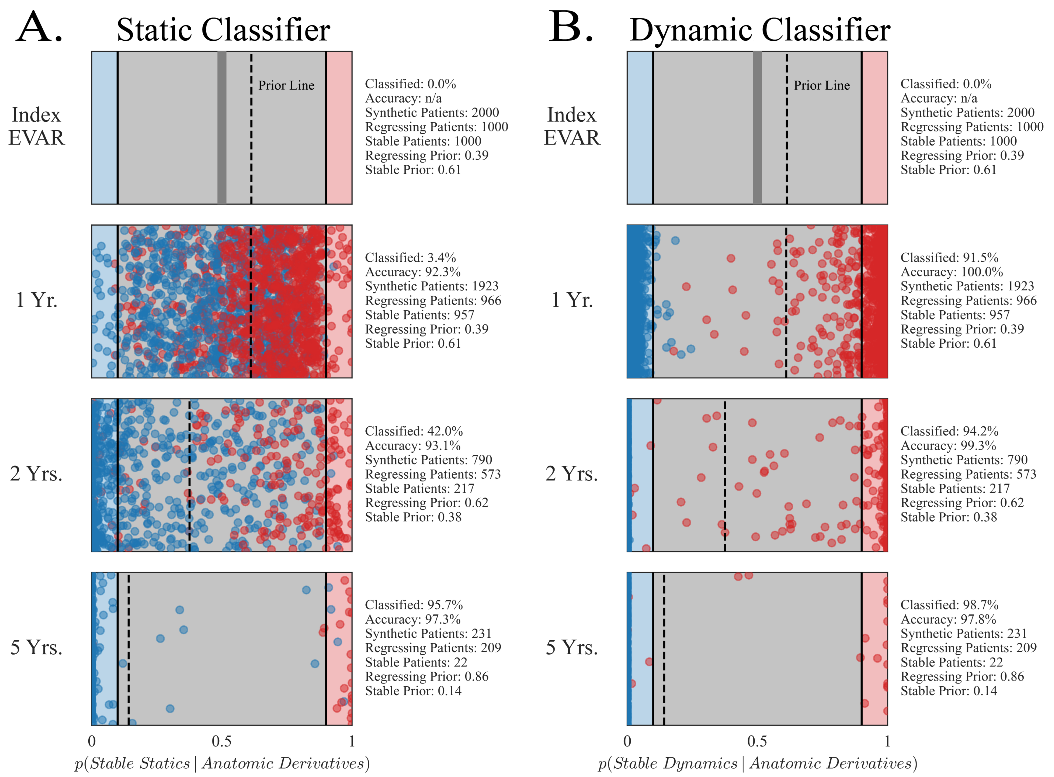

Figure 13: Posterior probabilities for static (A.) and dynamic (B.) Bayesian classifiers determined from simulated regressing and stable patient trajectories that proportionally follow the clinically-observed patient attrition rates shown observed in Fig. 12. Simulated trajectories experience randomized dropout corresponding to the fraction of patients remaining at that time point for each class, and the classifier priors are set to the proportion of remaining patients within each class. The dynamic classifier once again exits the "uncertain" region earlier than the static classifier, prior to the loss of the majority of patients, reflecting the added value of directional information in a clinically representative dataset. By maintaining this representativeness in both models' priors and the available proportions of data, we demonstrate the improved translational validity of probabilistic forecasts derived from dynamical models as compared to static models. This approach underscores how patients lost to follow-up shape evidentiary depth and should be explicitly accounted for in analyses of clinical datasets.
